# Treatment of Refractory Vesicourethral Anastomosis Pain Following Radical Prostatectomy Using a Combination of Non-ablative Erbium:YAG and Neodymium:YAG Laser Therapy: A Case Report

**DOI:** 10.7759/cureus.63036

**Published:** 2024-06-24

**Authors:** Nobuo Okui

**Affiliations:** 1 Dentistry, Kanagawa Dental University, Yokosuka, JPN

**Keywords:** refractory post-surgical pain, neodymium:yag laser, non-ablative erbium:yag laser therapy, radical prostatectomy, vesicourethral anastomosis pain

## Abstract

This case report describes the successful treatment of refractory vesicourethral anastomosis (VUA) pain in an 82-year-old man following radical prostatectomy using a combination of non-ablative erbium:YAG and neodymium:YAG laser therapy with Fotona SP Dynamis. Despite various conventional treatments, the patient's pain persisted, which significantly impaired his quality of life. The rationale for using laser therapy is based on its potential to promote tissue healing and nerve regeneration and reduce inflammation at the anastomosis site.

The patient underwent monthly laser irradiation sessions, with the erbium:YAG laser targeting the area around the urethral anastomosis site via the anus and the neodymium:YAG laser irradiating the base of the penis and scrotum. Urethral pain gradually decreased from a visual analog scale score of 10 to 0 over the course of treatment.

This highlights the importance of considering alternative approaches when conventional methods fail to provide relief. The targeted, minimally invasive nature of laser therapy may offer a safer and more effective alternative to systemic medications for managing chronic post-surgical pain. Although further research is needed to establish the generalizability and long-term effectiveness of this approach, this case provides a promising foundation for future investigations of the role of laser therapy in managing refractory VUA pain following radical prostatectomy.

## Introduction

Prostate cancer is one of the most prevalent malignancies in men worldwide [1]. Radical prostatectomy, a standard treatment for localized prostate cancer, often leads to post-operative complications such as vesicourethral anastomosis (VUA) pain [2]. VUA pain is a challenging and debilitating condition, significantly impairing patients' quality of life, as highlighted in previous studies [3]. Conventional treatments, including pharmacotherapy and nerve blocks, often fail to provide adequate relief in refractory cases of VUA pain. Various treatments, such as vacuum constriction devices, intraurethral and intracorporal alprostadil, and phosphodiesterase type 5 inhibitors, have been explored, but their effectiveness can vary widely due to differences in study design, data acquisition, and definitions of potency [4].

Recently, laser therapy has emerged as a promising alternative for the management of various lower urinary tract symptoms [5]. In particular, YAG lasers are solid-state lasers that utilize a crystal composed of yttrium and aluminum doped with erbium, known for their precise, controlled tissue heating without significant damage. Microablative CO_2_ lasers have been extensively investigated for the treatment of genitourinary syndrome of menopause (GSM) in breast cancer survivors (BCSs) [6-8]. Although the pathophysiology of GSM differs from that of VUA pain, the effectiveness of laser therapy in addressing refractory lower urinary tract symptoms suggests its potential application in managing VUA pain [9].

Moreover, recent studies have explored the use of laser therapy for male sexual dysfunction, highlighting the versatility of this approach in addressing urological conditions [10]. A combination of erbium:YAG and neodymium:YAG lasers has shown promise in improving erectile function and urinary incontinence in hemiplegic patients [11]. The tissue regeneration and vascularization effects of these lasers may also be beneficial for VUA pain management.

Furthermore, a case report of a 68-year-old woman with anal incontinence and vaginal atrophy successfully treated with non-ablative erbium:YAG and neodymium:YAG lasers demonstrated the potential of laser therapy in addressing complex pelvic floor disorders [12]. The concept of "re-canalization," which describes the restoration of blood flow in pelvic vessels through laser treatment, may be applicable to the management of VUA pain as well.

This case report describes an 82-year-old man with intractable VUA pain following radical prostatectomy. The patient underwent laparoscopic radical prostatectomy at the age of 75. At 78, he underwent cystostomy due to persistent perineal pain. Laser therapy was initiated at 79, and the pain gradually improved over a year. After two years of follow-up, the patient is now 82 years old. The rationale for this approach is discussed in light of the current evidence on vaginal laser therapy for GSM in BCSs as well as the emerging applications of laser therapy in male urological conditions. While acknowledging the limitations of a single case report, this study aimed to highlight the potential of laser therapy as a novel approach to managing refractory VUA pain and to stimulate further research in this area.

## Case presentation

A 75-year-old male patient underwent laparoscopic radical prostatectomy for prostate cancer (T2bN0M0). Surgery involved right nerve sparing, left nerve resection, and lymph node dissection. The bladder neck and urethra were anastomosed at six points using absorbable sutures. The estimated blood loss was 1200 ml, with 800 ml of autologous blood transfusion. Immediately after surgery, he experienced severe pain at the VUA site, which was described as deep in the urethra and perineal areas. The pain was exacerbated by sitting, urinary urgency, and the post-void period. Bowel movements caused severe pain at the anastomotic site, radiating to the lower abdomen and anus. Suspecting anastomotic stricture, the patient underwent endoscopic urethral stricture balloon dilation at 76 years of age; however, no improvement was observed. No chemotherapy or hormone therapy was administered throughout the treatment process.

Initially, the patient was managed with medication, as shown in Table 1. Non-opioid analgesics (acetaminophen) and non-steroidal anti-inflammatory drugs (NSAIDs; ibuprofen, loxoprofen, naproxen) were ineffective. Adjuvant medications for neuropathic pain (amitriptyline, pregabalin, duloxetine, etc.) were also tried but provided no relief, with pregabalin causing dizziness and duloxetine leading to psychiatric symptoms. A local anesthetic (lidocaine) was used for the sacral epidural block, but the effects were short-lived. Eventually, the patient required regular use of an opioid (fentanyl), which was effective, but caused severe side effects (nausea).

**Table 1:** Medications NSAIDs: Non-steroidal anti-inflammatory drugs

Category	Drug Name (Brand Name)	Characteristics	Effect
Non-opioid	Acetaminophen (Tylenol)	Effective for mild to moderate pain	No effect
NSAIDs	Ibuprofen (Brufen)	Has anti-inflammatory effect, effective for joint and muscle pain	No effect
NSAIDs	Loxoprofen (Loxonin)	Strong anti-inflammatory effect, effective for acute pain	No effect
NSAIDs	Naproxen (Naljclren)	Long-acting, effective for chronic pain	No effect
Adjuvants	Amitriptyline (Tryptanol)	Effective for neuropathic pain	No effect
Adjuvants	Pregabalin (Lyrica)	Effective for neuropathic pain and fibromyalgia	No effect. Causes dizziness
Adjuvants	Gabapentin (Gabapen)	Used for neuropathic pain	Not used
Adjuvants	Duloxetine (Cymbalta)	Effective for neuropathic pain and chronic musculoskeletal pain	No effect. Causes psychiatric symptoms
Local Anesthetic	Lidocaine (Xylocaine)	Used for localized pain	Sacral epidural block (short-term pain relief only)
Opioid	Fentanyl	Very potent, used for chronic pain and cancer pain	Effective but causes severe malaise

Several overactive bladder treatments have been attempted (Table 2). Anticholinergics (oxybutynin, tolterodine, and solifenacin), β3 agonists (mirabegron), antidepressants (imipramine), and topical medications (tolterodine/oxybutynin patch) were all ineffective and caused side effects, such as dry mouth, and mirabegron also induced arrhythmia.

**Table 2:** Overactive Bladder Medications

Category	Drug Name (Brand Name)	Side Effects	Effect
Anticholinergics	Oxybutynin (Detrusitol)	Dry mouth	No effect
Anticholinergics	Tolterodine (Detrusitol)	Dry mouth	No effect
Anticholinergics	Solifenacin (Vesicare)	Dry mouth	No effect
β3 agonist	Mirabegron (Betanis)	Dry mouth and inducing arrhythmia	No effect
Antidepressants	Imipramine (Tofranil)	Dry mouth	No effect
Topical medications	Tolterodine/Oxybutynin (Oxsroll Patch)	Dry mouth and skin irritation	No effect

Various procedures were performed to alleviate pain (Table 3). Physical therapy techniques (pelvic floor muscle exercises, electrical stimulation therapy, pelvic floor muscle massage), heat therapy (heating pad), cold therapy (ice pack), psychotherapy (cognitive behavioral therapy and counseling), and behavioral therapy (lifestyle modifications) were ineffective. Nerve block injections, specifically lidocaine (100 mg/10 mL) administration to the pudendal nerve and sacral epidural anesthesia, provided short-term relief lasting 1-3 hours.

**Table 3:** Ineffective Pain Management Procedures

Category	Procedure Name	Characteristics
Physical therapy	Pelvic floor muscle exercises	Strengthening and relaxation of pelvic floor muscles
	Electrical stimulation therapy	Uses electrical impulses to stimulate muscle contractions
Massage therapy	Pelvic floor muscle massage	Manual therapy to relax pelvic floor muscles
Heat therapy	Heating pad	Slight muscle tension relief. Provides warmth to the area
Cold therapy	Ice pack	Reduces inflammation and numbs the area
Psychotherapy	Cognitive behavioral therapy (CBT)	Changes thought patterns to manage pain
Counseling	Talk therapy	Provides emotional support and coping strategies
Behavioral therapy	Lifestyle modifications	Includes diet, exercise, and stress management changes
Nerve block	Nerve block injection	Lidocaine administration to pudendal nerve
Nerve block	Trigger point injection	Sacral epidural anesthesia
Surgical procedure	Cystostomy	Pain during urine storage was reduced, but no effect on pain caused by body movements

Due to the patient's severe pain, urodynamic testing, pressure-flow studies, and uroflowmetry could not be performed. The pain prevented the attachment of sensors for urodynamic testing and made it impossible to conduct pressure-flow studies. Additionally, the patient experienced severe pain and incontinence when a sudden urge to urinate occurred, rendering uroflowmetry unfeasible. At 78 years of age, it was predicted that urine accumulation would increase pressure on the urethral anastomosis site, exacerbating pain. Therefore, cystostomy was performed. This intervention reduced severe perineal pain associated with urinary urgency based on a visual analog scale (VAS) score of 10-5. However, pain deep in the urethra and perineal area, triggered by body movements such as sitting, remains unresolved. The high-sensitivity prostate-specific antigen was less than 0.001 ng/mL.

**Figure 1:**
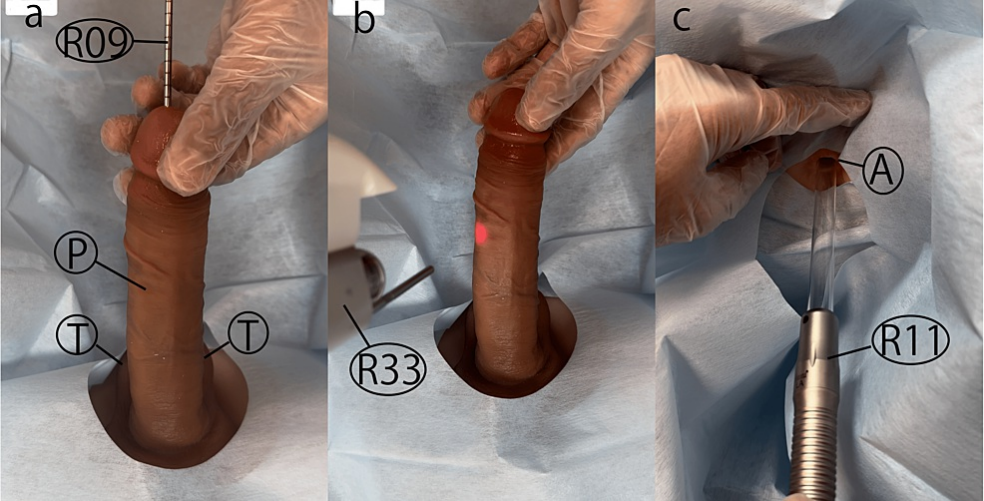
The Three Laser Treatment Approaches a: UEL step (whole urethral laser irradiation with R09), b: Nd:YAG step (Nd:YAG laser PIANO mode with R33, c: AEL or REL step (inserting the handpiece into the anus or rectum) P: Penis, T: Testes, R: Rectum, A: Auns, R09: whole urethral laser irradiation, R33: R33 non-contact handpiece, R11: R33 non-contact handpiece.

Figure 1 shows that based on previous studies, three laser treatment methods were considered: (1) inserting the handpiece into the urethra (UEL) [13], (2) irradiating the skin surface with an Nd:YAG laser [10-12], and (3) inserting the handpiece into the anus or rectum (AEL or REL) [10-12]. However, method (1) could not be performed due to the severe pain in the urethra. Therefore, the following two methods were adopted.​​

Fotona SP Dynamis (Fotona d.o.o., Ljubljana, Slovenia) was selected. REL uses VEL with a circular adapter and R11 handpiece at 3.00 J/cm², 2.0 Hz, and 7 mm spot sizes, covering the entire vagina in six segments once a month. 

The Nd:YAG step was administered using a 1064 nm Nd:YAG laser in a continuous PIANO pulse mode for a duration of 30 minutes, utilizing a non-contact R33 handpiece set at an energy density of 90 J/cm², with a five-second pulse duration, and a 9 mm spot size, targeting the penis and scrotum.

**Figure 2:**
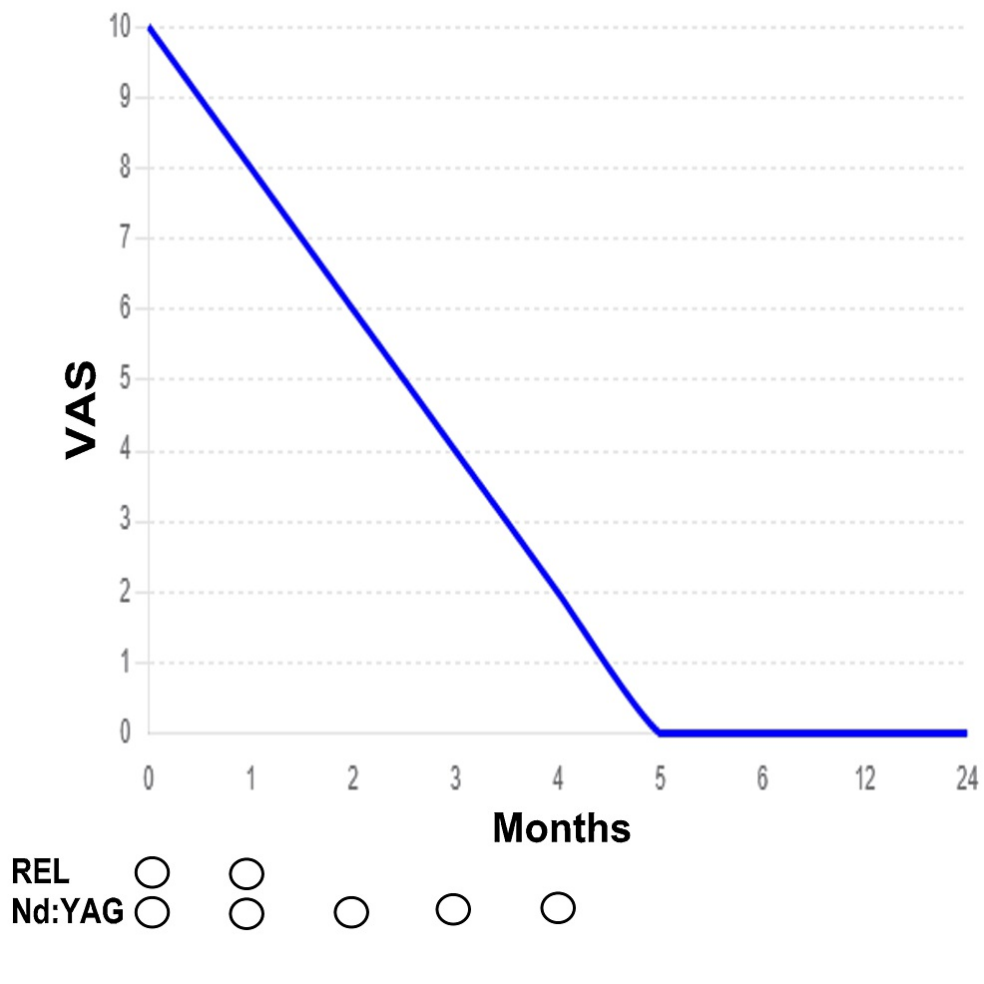
Reduction in Urethral Pain With Nd:YAG Laser and REL Treatment Y-axis: VAS (visual analog scale), where 0 indicates no pain and 10 represents maximum pain; X-axis: Time progression (in months) AEL: Non-ablative erbium laser treatment for the anus; Nd:YAG: Neodymium laser treatment for the penis and surrounding areas Circles (◯) indicate treatment sessions (REL: two sessions, Nd:YAG: five sessions)

With monthly irradiation sessions, the urethral pain decreased from a VAS score of 10 to 8, 6, 4, 2, and finally 0, as shown in Figure 2. The anal pain disappeared after the second session, so the REL treatment was discontinued. In total, the patient received five sessions of Nd:YAG laser treatment, with the first two sessions combined with REL and the remaining three sessions using only Nd:YAG laser. The pain associated with urine accumulation in the bladder could be alleviated by adjusting the urine volume using a catheter inserted into the bladder fistula. However, before the treatment, the catheter needed to be frequently opened, which interfered with daily life. As the laser treatment progressed, the pain during urine storage reduced in proportion to the improvement of urethral pain. When the urethral pain reached 0, the patient was able to store 300 ml of urine in the bladder. The patient has remained pain-free for two years up to the present day.

**Figure 3:**
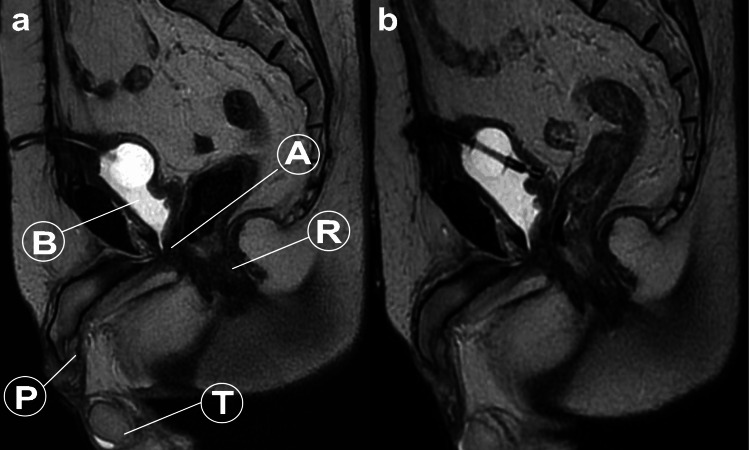
MRI Sagittal Images Before and After Treatment B: Bladder, P: Penis, T: Testes, R: Rectum, A: Anastomosis

Fentanyl was discontinued when the VAS score reached 8, and loxoprofen was discontinued when the VAS score was 4. Currently, the patient does not require NSAIDs and reports no pain (VAS score of 0). Figure 3 shows MRI images reconstructed to sagittal and axial views with an 8-mm thickness after maximum intensity projection treatment. These images were obtained before (T0) and after (T18) the laser treatment. No significant changes were observed between the pre-treatment and post-treatment scans. It is hypothesized that subtle changes, which may not be detectable on MRI, may have contributed to the alleviation of nerve pain at the anastomosis site following laser treatment.

**Figure 4:**
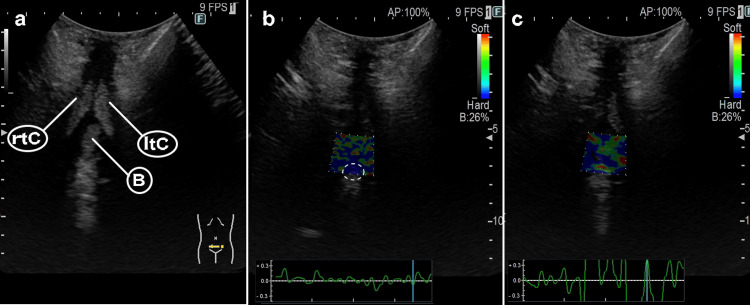
Ultrasound Elastography of the Peri-urethral Tissue Before and After Treatment (a) Anatomical diagram before setting the elasticity mode, showing the bulbar urethra (B), right corpora cavernosa (rtC), and left corpora cavernosa (LtC). (b) Elasticity imaging at pre-treatment timepoint T0, revealing partially stiff tissue in the bulbar urethra. In the area enclosed by the white dotted line, the center of the hypoechoic region exhibits strain, while the surrounding area shows confirmed strain, corresponding to a Tsukuba Elasticity Score of 3. (c) Elasticity imaging at timepoint T18, demonstrating more uniformly soft tissue in the bulbar urethra compared to the pre-treatment timepoint. The entire region lacks hypoechoic areas and shows no strain, equivalent to a Tsukuba Elasticity Score of 5.

Tissue stiffness was investigated by ultrasound (Arietta65, Fujifilm Co., Tokyo, Japan). However, since normal values for tissue stiffness have not been reported in previous studies, these findings are considered referential [14]. By setting the ultrasound device to elasticity mode and adjusting the probe for deep measurement, the bulbar urethra can be visualized by placing the probe horizontally along the lower edge of the pubic bone. Figure 4a shows an anatomical diagram before setting the elasticity mode, where B, rtC, and LtC represent the bulbar urethra, right corpora cavernosa, and left corpora cavernosa, respectively. The corpora cavernosa, which constitutes the majority of the penis, is a symmetrical hypoechoic oval-shaped structure on both sides of the bulbar urethra. The bulbar urethra, a part of the urethra, is located just below the root of the penis and is depicted as a central hypoechoic tubular structure. Although not visible on the screen, the prostate is normally visualized behind the bulbar urethra, and an anastomosis site was present in this patient. In this patient, the root cause of the pain was the anastomosis site. As shown in Figure 4b, at the pre-treatment time point T0, the bulb was not uniformly soft on elasticity imaging because of this influence and was confirmed to be partially stiff tissue. On the other hand, at T18, shown in Figure 4c, the bulb appears to be a more uniform soft tissue with less strain on elasticity imaging compared to the previous timepoint.

Regarding elasticity, Fujifilm established standards by using breast tissue as a sample [14]. According to these standards, Tsukuba Elasticity Score 1 indicates strain throughout the entire hypoechoic area, Score 2 indicates an absence of strain in part of the hypoechoic area, Score 3 indicates strain in the marginal zone without strain in the center of the hypoechoic area, Score 4 indicates strain throughout the entire hypoechoic area, and Score 5 indicates a complete absence of strain [14]. Scores 1 and 2 are important indicators for diagnosing breast cancer, while Score 3 signifies inflammation or scarring. In the current images, Figure 4b shows that in the area enclosed by the white dotted line, the center of the hypoechoic region exhibits strain, whereas the surrounding area shows a confirmed strain, corresponding to a Tsukuba Elasticity Score of 3, capturing the area around the anastomosis site after prostatectomy. In contrast, Figure 4c demonstrates that the entire region lacks hypoechoic areas and shows no strain, equivalent to a Tsukuba Elasticity Score of 5. During the laser treatment, no side effects such as infections or increased pain were observed.

## Discussion

In this report, we describe the case of an 82-year-old man who suffered from intractable VUA following radical prostatectomy. Despite various conventional treatments including pharmacotherapy and nerve blocks, the patient's pain persisted, significantly impairing his quality of life. The successful management of his pain using a combination of non-ablative erbium:YAG and neodymium:YAG laser therapy highlights the potential of this novel approach for addressing refractory VUA pain.

One of the key points to consider in this case is that pain may not always be detectable by MRI. In particular, when minute nerve entrapment occurs at the anastomosis site following radical prostatectomy, identifying the source of pain can be challenging [2,3]. This emphasizes the importance of considering alternative diagnostic and therapeutic approaches when conventional imaging modalities fail to provide a clear explanation of the patient's symptoms.

Moreover, the existing pain management guidelines may not adequately address the unique challenges posed by VUA pain. Although guidelines for cancer-related pain are well established [1], they may not be applicable to patients who have undergone curative surgery and are experiencing post-surgical pain. Furthermore, chronic pain guidelines often rely on pharmacological interventions [4], which can lead to systemic side effects, as seen in this patient who suffered from the adverse effects of fentanyl and overactive bladder medications.

The successful use of localized laser therapy in this case underscores the potential benefits of targeted and minimally invasive approaches for managing refractory VUA pain. Laser therapy may provide a safer and more effective alternative to systemic medications by focusing on a specific area of pain and promoting tissue healing through photobiomodulation [5-9]. The non-ablative nature of the Er:YAG and Nd:YAG lasers used in this case may have contributed to their efficacy and safety, as they promote tissue regeneration without causing surface damage [10,11].

In this case, the rationale for using a combination of lasers was based on their complementary effects. The erbium:YAG laser targets superficial tissues, promoting collagen remodeling and neovascularization [5,6], whereas the neodymium:YAG laser penetrates deeper, reaching the periprostatic neurovascular bundles [10,11]. By addressing both superficial and deep structures, this combined approach may have contributed to the patient's successful pain relief.

Although the exact mechanisms underlying the effectiveness of laser therapy in this case remain to be elucidated, it is plausible that the treatment promoted nerve regeneration and reduced inflammation at the anastomosis site [9]. The gradual improvement in pain scores over multiple treatment sessions suggests a cumulative effect possibly due to progressive tissue healing and nerve regeneration.

In addition to the clinical findings, this case report presents preliminary ultrasound elastography data that may provide insights into the changes in tissue stiffness associated with VUA pain and its treatment. The pre-treatment image (Figure 4b) suggests increased tissue stiffness around the anastomosis site, whereas the post-treatment image (Figure 4c) shows a reduction in stiffness following laser therapy. However, it is important to emphasize that ultrasound elastography is not yet an established diagnostic tool for VUA pain and that these findings should be interpreted with caution [14].

The findings of this case report are consistent with those of recent studies investigating the use of laser therapy for various post-surgical and oral pain conditions. A meta-analysis by Sales et al. found that the erbium:YAG laser was effective in reducing edema, pain, and complications following removal of impacted mandibular third molars [15]. Although this study focused on a different surgical procedure, the results support the potential of erbium:YAG laser therapy in managing post-surgical pain and promoting tissue healing.

Similarly, a comparative study by Rocca et al. investigated the effect of four different laser wavelengths on pain management in recurrent aphthous stomatitis (RAS) [16]. The study found that diode lasers at 808 nm, 450 nm, and 635 nm provided significant pain relief, with the 635 nm laser showing the earliest effect. Although RAS is a different condition from VUA pain, this study highlights the potential of laser therapy in managing oral pain and promoting tissue healing.

These studies, along with a case report by Gaspar et al. [13] on the use of non-ablative erbium:YAG laser therapy for chronic prostatitis/chronic pelvic pain syndrome, provide further evidence supporting the potential of laser therapy in managing various types of pain and promoting tissue regeneration. Although the specific conditions and laser parameters may differ, the underlying mechanisms of photobiomodulation and tissue regeneration appear to be consistent across these studies.

In addition to the clinical findings, this case report presents preliminary ultrasound elastography data that may provide insights into the changes in tissue stiffness associated with VUA pain and its treatment. Ultrasound elastography has been used in previous research to assess breast tissue stiffness for breast cancer screening [14]. In the current case, the pretreatment image (Figure 4b) suggests increased tissue stiffness around the anastomosis site, whereas the post-treatment image (Figure 4c) shows a reduction in stiffness following laser therapy. However, it is important to emphasize that ultrasound elastography is not yet an established diagnostic tool for VUA pain and that these findings should be interpreted with caution. Further research is needed to validate the use of ultrasound elastography to assess tissue stiffness changes associated with VUA pain and treatment.

Although this case report provides evidence for the potential of laser therapy in managing refractory VUA pain, it is important to acknowledge its limitations. As a single case, these findings may not be generalizable to all patients with VUA pain. Additionally, the lack of long-term follow-up data limits our understanding of the durability of treatment effects. Further research, including randomized controlled trials with larger sample sizes and longer follow-up periods, is required to validate the efficacy and safety of this approach.

## Conclusions

This case report demonstrates the successful use of a combination of non-ablative erbium:YAG and neodymium:YAG laser therapy in managing refractory VUA pain following radical prostatectomy. This highlights the importance of considering alternative diagnostic and therapeutic approaches when the conventional methods fail to provide relief. The targeted, minimally invasive nature of laser therapy may offer a safer and more effective alternative to systemic medications in managing chronic post-surgical pain. The findings of this case report are supported by recent studies investigating the use of laser therapy in various post-surgical and oral pain conditions, underscoring the potential of this approach in promoting tissue healing and managing pain. Although further research is needed to establish the generalizability and long-term effectiveness of this approach, this case provides a promising foundation for future investigations into the role of laser therapy in managing refractory VUA pain.

## Appendices

Detailed medication history

The patient's medication history is complex and involves various dosages, durations, and multiple prescribers. Ibuprofen was prescribed at 1200 mg per day for three months and 3200 mg per day for one month. Loxoprofen was prescribed at 60 mg per day for one month and 180 mg per day for six months. Additionally, the patient self-administered over-the-counter loxoprofen at 360 mg per day for several days without the physician's knowledge. Naproxen was prescribed at 500 mg per day for one month, 1000 mg per day for two months, and 1500 mg per day for six months by physicians outside our institution. It is important to note that ibuprofen and loxoprofen were simultaneously prescribed for several months by an unknown physician.

Amitriptyline was prescribed at 150 mg per day for four months. Pregabalin was initially prescribed at 150 mg per day for two months, followed by 600 mg per day for several months, which was prescribed by a psychiatrist without our institution's knowledge. Gabapentin was estimated to have been prescribed at 600 to 800 mg per day for at least two months. Duloxetine was prescribed at 60 mg per day for one month and 120 mg per day for three months; however, the patient's compliance was low due to the simultaneous administration of multiple medications and prescriptions from multiple physicians. Fentanyl was initially prescribed at the typical dosage of 25 mcg/hour patch, changed every 72 hours. However, it was discontinued after a few days due to side effects. Despite this, the patient continued to seek and receive fentanyl prescriptions from physicians outside our institution.

Detailed overactive bladder medication history

The patient's overactive bladder treatment history involves various medications, dosages, and durations. Oxybutynin (Detrusitol) was prescribed at 5 mg daily for one month, while tolterodine (Detrusitol) was prescribed at 4 mg daily for one month. Solifenacin (Vesicare) was prescribed at 5 mg daily for one month, followed by 10 mg daily for three months. Mirabegron (Betanis), a β3 agonist, was prescribed at 25 mg daily for one month and then increased to 50 mg daily for three months. Imipramine (Tofranil), an antidepressant, was prescribed at 25 mg daily for one month. A topical medication, tolterodine/oxybutynin (Oxsroll Patch), was prescribed at 3.9 mg/24 hours for one month. It is important to note that the patient was simultaneously prescribed Detrusitol 4 mg and Betanis 25 mg for three months, followed by Detrusitol 4 mg and Betanis 50 mg for another three months.

Pain management procedures

The patient underwent various pain management procedures, including physical therapy techniques, psychotherapy, behavioral therapy, and nerve block injections. Physical therapy techniques, such as pelvic floor muscle exercises, electrical stimulation therapy, and pelvic floor muscle massage, were performed to strengthen and relax the pelvic floor muscles. However, these techniques did not provide significant relief for the patient's pain. Psychotherapy, including cognitive behavioral therapy and counseling, was administered by mental health professionals to help the patient manage pain and cope with the emotional impact of the condition. Due to privacy concerns and the group-based nature of these interventions, detailed information on the patient's participation and progress could not be obtained. Behavioral therapy, focusing on lifestyle modifications such as diet, exercise, and stress management, was also implemented. However, these changes did not result in substantial pain relief for the patient. Nerve block injections, specifically lidocaine administration to the pudendal nerve and sacral epidural anesthesia, provided short-term relief lasting 1-3 hours. The limited duration of pain relief highlights the challenges in managing the patient's chronic pain condition.
